# Associations of Biomarkers of Systemic Inflammation, Angiogenesis, and Cell‐to‐Cell Adhesion With Tumor Budding Among Early‐Onset and Later‐Onset Colorectal Cancer Patients

**DOI:** 10.1002/cam4.71267

**Published:** 2025-09-23

**Authors:** Oda Hausmann, Pauline P. Schobert, Jennifer Ose, Caroline Himbert, Maria Pletneva, Jolanta Jedrzkiewicz, Anne Nguyen, Tengda Lin, Christy A. Warby, Sheetal Hardikar, Anita R. Peoples, Ildiko Strehli, Lyen C. Huang, Jessica N. Cohan, Bartley Pickron, Courtney Scaife, Christopher I. Li, William M. Grady, David Shibata, Adetunji T. Toriola, Martin Schneider, Jane C. Figueiredo, Erin M. Siegel, Biljana Gigic, Stephan Herzig, Mmadili N. Ilozumba, Cornelia M. Ulrich

**Affiliations:** ^1^ Huntsman Cancer Institute Salt Lake City Utah USA; ^2^ Department of Population Health Sciences University of Utah Salt Lake City Utah USA; ^3^ Technical University of Munich Munich Germany; ^4^ Ludwig‐Maximilians‐University Munich Germany; ^5^ Department of Information and Communication, Faculty for Media, Information and Design University of Applied Sciences and Arts Hannover Germany; ^6^ Clinical and Translational Epidemiology Unit Massachusetts General Hospital and Harvard, Medical School Boston Massachusetts USA; ^7^ Department of Epidemiology Harvard T. H. Chan School of Public Health Boston Massachusetts USA; ^8^ Department of Pathology University of Utah Salt Lake City Utah USA; ^9^ Department of Surgery University of Utah Salt Lake City Utah USA; ^10^ Fred Hutchinson Cancer Center Seattle WA USA; ^11^ University of Washington School of Medicine Seattle WA USA; ^12^ University of Tennessee Health Science Center Memphis Tennessee USA; ^13^ Division of Public Health Sciences, Department of Surgery Washington University School of Medicine St. Louis Missouri USA; ^14^ University Hospital Heidelberg, Department of Surgery Heidelberg Germany; ^15^ Department of Medicine, Samuel Oschin Comprehensive Cancer Center Cedars Sinai Medical Center Los Angeles California USA; ^16^ Department of Cancer Epidemiology H. Lee Moffitt Cancer Center and Research Institute Tampa Florida USA; ^17^ National Center for Tumor Diseases (NCT) Heidelberg Germany; ^18^ Institute for Diabetes and Cancer IDC Helmholtz Center Munich Munich Germany; ^19^ Joint Heidelberg‐IDC Translational Diabetes Program Heidelberg University Hospital Heidelberg Germany; ^20^ Molecular Metabolic Control Technical University Munich Munich Germany

**Keywords:** colorectal cancer, CRP, early‐onset colorectal cancer, IL‐8, sICAM‐1, systemic inflammation, tumor budding

## Abstract

**Background:**

High tumor budding and elevated systemic inflammation are adverse prognostic indicators in colorectal cancer. Its underlying mechanisms remain poorly understood. It is unclear whether systemic inflammation, angiogenesis, and cell‐to‐cell adhesion influence tumor budding.

**Methods:**

We investigated *n* = 132 stage I–III colorectal cancer patients recruited at Huntsman Cancer Institute enrolled in the ColoCare Study. Tumor budding was evaluated using an evidence‐based scoring system, and patient sera were analyzed for nine circulating biomarkers using the Meso Scale Discovery platform. We examined associations between biomarkers and tumor budding using multivariable linear regression models adjusted for age, sex, neoadjuvant treatment, stage, and non‐steroidal anti‐inflammatory drug use.

**Results:**

The study population was predominantly non‐Hispanic White (95%), with a mean age of 61 years; 56% were male. Most tumors were stage III (47%), located in the colon (64%), and exhibited low‐grade tumor budding (58%). Soluble intercellular adhesion molecule 1 was inversely associated with tumor budding overall (M1: *β* = −0.57, *p* = 0.03), among females (M1: *β* = −0.81, *p*‐value = 0.03) and later‐onset (≥ 50 years) colorectal cancer (M1: *β* = −0.71, *p*‐value = 0.008). C‐reactive protein was positively associated with tumor budding in males (M1: *β* = 0.23, *p* = 0.001), while interleukin‐8 (M1: *β* = 0.96, *p*‐value = 0.01) and soluble vascular adhesion molecule 1 (M2: *β* = 1.48, *p*‐value = 0.04) were positively associated with tumor budding in early‐onset patients. However, these associations did not remain statistically significant after correction for multiple testing.

**Conclusion:**

Overall, our findings do not provide evidence of a significant association between biomarkers of systemic inflammation, angiogenesis, and cell‐to‐cell adhesion with tumor budding count. We observed patterns for some biomarkers, yet none remained statistically significant after correction for multiple testing. These findings provide preliminary insights for future studies.

**Trial Registration:** ClinicalTrials.gov: NCT02328677.

## Graphical abstract

This study identified patterns between biomarkers of systemic inflammation, angiogenesis, and cell‐to‐cell adhesion with tumor budding in colorectal cancer, suggesting differences by age of disease onset and sex. These findings contribute to our understanding of the underlying mechanism of tumor budding and may provide guidance for future studies investigating the biological mechanisms of tumor budding that may be relevant for prognostic assessments and therapeutic interventions in colorectal cancer.

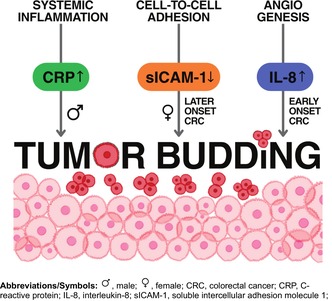

**Abbreviations**
ANOVA: one‐way analysis of variance
CAP: College of American Pathologists
CRP: C‐reactive protein
CV: coefficients of variation
EMT: epithelial‐to‐mesenchymal transition
HCI: Huntsman Cancer Institute
IL‐6: interleukin‐6
IL‐8: interleukin‐8
M1: Model 1 (age‐ and sex‐adjusted)
M2: Model 2 (multivariable adjusted)
NSAIDs: non‐steroidal anti‐inflammatory drugs
SAA: serum amyloid A
sICAM‐1: soluble intercellular adhesion molecule 1
sVCAM‐1: soluble vascular adhesion molecule 1
TNF‐α: tumor necrosis factor alpha
VEGF‐A: vascular endothelial growth factor A
VEGF‐D: vascular endothelial growth factor D

## 1 Introduction

Colorectal cancer is the third most common type of cancer, with 1.8 million new cases diagnosed globally each year [1]. Although the incidence rate of later‐onset colorectal cancer (≥ 50 years) has declined, there has been an alarming increase in early‐onset colorectal cancer (< 50 years) [2]. The underlying mechanisms driving early‐onset colorectal cancer remain largely unknown [3]. High‐grade tumor budding is an important prognostic factor in colorectal cancer [4, 5, 6, 7]. Tumor budding is defined as the presence of small clusters of tumor consisting of up to four malignant cells, located at the advancing edge during histopathologic evaluation of colonic adenocarcinoma [8]. Tumor budding is an independent predictor of overall and disease‐free survival and colorectal cancer recurrence [8]. It is closely linked to other prognostic factors such as higher tumor grade and stage, positive nodal status, lympho‐vascular invasion, infiltrating‐type invasive tumor margin, left‐sided tumor localization, mismatch‐repair proficiency, and distant metastases [8]. In a clinical setting, tumor budding plays a crucial role in the decision‐making process regarding whether to proceed with surgical resection, such as colectomy after the diagnosis of a primary tumor [9]. In the context of stage II cancers, tumor budding can significantly impact the decision of whether to administer chemotherapy or not [9]. Clinical guidelines by the College of American Pathologists (CAP) recommend reporting tumor budding count for colorectal cancer arising in polyps and stage I–II as part of clinical care [10]. Despite the clinical relevance of tumor budding, the underlying biological mechanism of its association with poor clinical outcomes remains unclear [11].

Systemic inflammation, a hallmark of cancer, also plays an important role in cancer progression and has been associated with poor prognosis in colorectal cancer [12]. It can lead to the activation of immune cells and the release of cytokines, growth factors, and other signaling molecules that promote angiogenesis, tissue invasion, and metastasis [12]. Systemic inflammation can be measured with biomarkers such as C‐reactive protein (CRP) [13], serum amyloid A (SAA) [14], interleukin‐6 (IL‐6) [15], and interleukin‐8 (IL‐8) [16]. In addition, biomarkers of cell‐to‐cell adhesion and angiogenesis such as soluble intercellular adhesion molecule 1 (sICAM‐1) [17], soluble vascular adhesion molecule 1 (sVCAM‐1) [17], tumor necrosis factor alpha (TNF‐α) [18], and vascular endothelial growth factor A (VEGF‐A) and vascular endothelial growth factor D (VEGF‐D) [19] are commonly assessed.

Previous studies have described epithelial‐to‐mesenchymal transition (EMT) as the histomorphological correlate to tumor budding [11, 20, 21]. EMT is a mechanism that allows tumor cells to gain a malignant phenotype, enabling them to colonize distant organs. EMT is characterized by the loss of tumor cell polarity and adhesion, increased motility, and evasion of apoptosis [11]. Preclinical studies support a role of systemic inflammation in EMT [22, 23, 24, 25, 26, 27, 28]. For example, a colorectal cancer mouse model demonstrated that IL‐6 signaling induced EMT [25]. The study uncovered a feedback loop, which involves the promotion and maintenance of EMT [25]. Another study suggested that a reduction of EMT and invasive/migratory abilities of colorectal cancer cells was observed upon neutralizing IL‐6 and IL‐8 [22]. In a preclinical study using colorectal cancer cells, it was demonstrated that TNF‐α regulates EMT, resulting in enhanced metastases of colorectal cancer cells [23]. Furthermore, Kudo et al. indicated that CRP suppresses EMT in colorectal cancer cells through the inhibition of N‐cadherin and ZEB‐1 [27].

**FIGURE 1:**
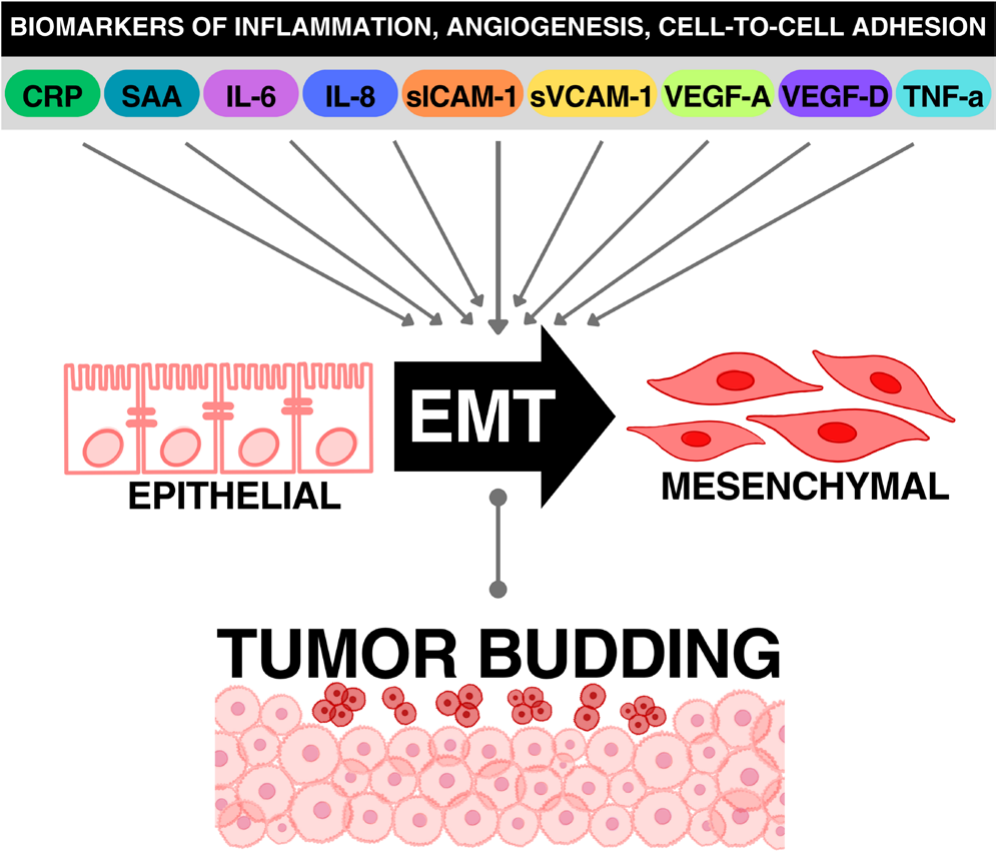
Relationship between biomarkers of inflammation/angiogenesis/cell‐to‐cell adhesion, EMT, and tumor budding. EMT, epithelial‐to‐mesenchymal transition.

While these studies demonstrate a link between biomarkers of inflammation/angiogenesis/cell‐to‐cell adhesion and EMT, there is a critical gap in our knowledge regarding the relationship between biomarkers of these biological processes and tumor budding. The primary objective of this study was to examine the associations of biomarkers of inflammation, angiogenesis, and cell‐to‐cell adhesion with tumor budding in colorectal cancer patients. We hypothesized that higher biomarker levels of inflammation, angiogenesis, and cell‐to‐cell adhesion would be associated with higher tumor budding counts since tumor budding and most biomarkers have been positively associated with EMT in previous research (Figure 1) [22, 23, 24, 25, 26, 27, 28]. To the best of our knowledge, this is the first study evaluating the link between systemic inflammation and tumor budding in colorectal cancer.

## 2 Materials and Methods

### 2.1 Study Population

The present study is conducted as part of the prospective ColoCare Study (ClinicalTrials.gov Identifier: NCT02328677), an international cohort of newly diagnosed stage I–IV colorectal cancer patients (ICD‐10 C18–C20) [29]. The ColoCare Study is a U01‐funded multicenter study of transdisciplinary research on colorectal cancer outcomes and prognosis [29]. ColoCare Study inclusion criteria are: patients newly diagnosed with colon or rectal cancer (stages I–IV), age ≥ 18 years, English (U.S. sites) or German (German site) speaking, and mentally/physically able to consent and participate. Patients who meet the inclusion criteria are recruited for the ColoCare Study prior to surgical treatment. Baseline examination includes anthropometric measurements, biospecimen collection (e.g., blood, stool, urine, saliva, fresh frozen tumor, and normal tissue), and self‐administered questionnaires on symptoms, health behaviors, and health‐related quality of life.

This study included *n* = 132 patients with stage I–III colorectal cancer, who had pre‐surgery biomarker measurements and hematoxylin and eosin (H&E) stained colorectal tumor sections for tumor budding count available at Huntsman Cancer Institute (HCI). The study received approval from the Institutional Review Board at the University of Utah, and all participants granted their written informed consent.

### 2.2 Blood Processing and Biomarker Assays

Blood samples were obtained prior to surgery and at least 2 weeks after completion of neo‐adjuvant treatment, if administered. Serum was extracted and stored at −80°C in 110 μL aliquots. Assays were run using the Mesoscale Discovery Platform (MSD, Rockville, MD, USA) in the Ulrich laboratory at HCI as previously described [30, 31, 32, 33, 34, 35]. All samples were blinded, run in duplicate, and analyzed using the MSD Sector Imager 2400A. The VEGF‐A and VEGF‐D assays were run on a V‐Plex Angiogenesis Panel Human Kit at a dilution of 1:8, while IL‐6, IL‐8, and TNF‐α were run on a V‐Plex Proinflammatory Panel 1 Human Kit at a dilution of 1:2, and CRP, SAA, sICAM‐1, and sVCAM‐1 were run on a V‐Plex Vascular Injury Panel 2 Human Kit at a dilution of 1:2500. Each plate included three quality controls to calculate inter‐ and intra‐plate coefficients of variation (CV). The inter‐plate CV was below 9.6% for all batches, and the intra‐batch CV was 3.9%. The selected biomarker panel was based on previous studies that investigated central biomarkers for inflammation/angiogenesis/cell‐to‐cell adhesion molecules and behavioral outcomes in both the general and cancer population [30, 31, 32, 33, 34, 35, 36, 37, 38, 39].

### 2.3 Assessment of Tumor Budding in Tumor Tissue

The University of Utah Department of Pathology provided H&E‐stained colorectal cancer tissue slides collected during surgical resection of the primary tumor. The presence of tumor budding, defined as a single tumor cell or a cluster of up to four tumor cells at the advancing tumor edge, was evaluated by two participating board‐certified pathologists (JJ and MP) in accordance with the recommendations provided by the international tumor budding consensus conference [8, 10].

Tumor budding was assessed on a single representative slide per patient, with counts performed in the hotspot area after review of all available tumor slides. The “hotspot” method was used to quantify tumor budding on H&E‐stained slides, with 10 individual fields scanned at medium power (10× objective) to ensure standardized field size (0.785 mm^2^). Tumor budding scores were reported by area (mm^2^) rather than objective lens. Tumor budding scores were considered on a continuous scale and in the descriptive analyses further categorized into clinical categories (low, intermediate, high) and tertiles.

### 2.4 Statistical Analysis

Due to right‐skewed distributions, tumor budding and biomarker values were log2‐transformed to enable normal distribution. One‐way analysis of variance (ANOVA) and Chi‐square tests of independence were used to compare the characteristics of the study participants by tumor budding count in tertiles and clinical categories for continuous and categorical variables, respectively. We decided to utilize tertiles to categorize tumor budding, rather than the clinical distinction due to the limited number of clinically high tumor budding cases in our study (14%). Tertiles allowed us to distribute the data more evenly, enhancing the statistical robustness of our analyses. Biomarkers of inflammation, angiogenesis, and cell‐to‐cell adhesion were the independent variables, while tumor budding was the dependent variable. Multivariable linear regression was used to investigate the associations between log2‐transformed biomarker values and log2‐transformed tumor budding counts.

The following variables were evaluated and examined for confounding: age at diagnosis (< 50/≥ 50 years), sex (female/male), neoadjuvant treatment (no/yes), race (White/non‐White), tumor location (colon/rectum), tumor grade [1, 2, 3], tumor stage (I, II, III), body mass index (BMI, ≥ 18.5 and < 25 kg/m^2^; ≥ 25 and < 30 kg/m^2^; ≥ 30 kg/m^2^), smoking status (non‐smoker, former smoker, current smoker), non‐steroidal anti‐inflammatory drug (NSAID) use at least once per week over the past year (no, yes/aspirin or aspirin plus non‐aspirin NSAIDs, yes/non‐aspirin), and scoring pathologist (JJ/MP). Model 1 (M1) was adjusted for age and sex. Model 2 (M2) was developed using a stepwise selection procedure, adjusting for age, sex, neoadjuvant treatment, stage, and NSAIDs. Variables were included or retained based on a significance level of 0.15.

The subgroup analysis was based on a priori hypotheses. We conducted stratified analyses to determine whether associations between biomarkers of inflammation, angiogenesis, and cell‐to‐cell adhesion and tumor budding were modified by age (early‐onset colorectal cancer [< 50 years] and later‐onset colorectal cancer [≥ 50 years]) or sex (male vs. female). Considering the relatively small number of early‐onset colorectal cancer patients (*n* = 22), we also conducted stratified analysis using an alternative cut‐off of < 60 years vs. ≥ 60 years. Although blood samples were taken at least 2 weeks after neo‐adjuvant treatment, we performed sensitivity analyses in which we excluded all patients (*n* = 32) who had ever received neo‐adjuvant treatment. Statistical significance was defined as nominal *p* < 0.05, and all statistical tests were 2‐sided. Since this analysis focused on pre‐hypothesized associations, we present results adjusted for age and sex; multivariable adjustment and the Bonferroni adjustment. We accounted for multiple testing using the Bonferroni correction for testing 9 biomarkers with the alpha level set at *p* = 0.01 (0.05/9). Analyses were performed using SAS version 9.3 (SAS Institute Inc.).

## 3 Results

Demographic and clinicopathological patient characteristics of the study population (*n* = 132) are presented in Table 1. Patients had an average age of 61 years, 17% of the patients were diagnosed with early‐onset colorectal cancer, 56% were males, and 95% identified as White. Most tumors (64%) were colonic and 47% were diagnosed as stage III cancer. Most patients (77%) did not receive neoadjuvant treatment and had moderately differentiated tumors (64%). 52% were considered inactive and 75% overweight (≥ 25 and < 30 kg/m^2^) or obese (≥ 30 kg/m^2^). 45% were never smokers, and 42% reported regular NSAID use (at least once per week) during the past year. Tumor budding count was reported with a mean of 5 tumor buds by area (SD = 4.21; range = 0–23) and the majority of patients (58%) were categorized with low‐grade tumor budding (low‐grade: 0–4 buds) followed by intermediate‐grade budding (5–9 buds) (28%).

**TABLE 1:** Baseline demographic and clinicopathologic baseline characteristics of individuals with invasive colorectal cancer (*n* = 132).

Age (years)	
Mean ± SD	61 ± 13
Median (IQR)	61 (52–71)
Age, *n* (%)	
Early‐onset (< 50 y)	22 (17)
Later‐onset (≥ 50 y)	110 (83)
Sex, *n* (%)	
Female	58 (44)
Male	74 (56)
Race, *n* (%)	
White	125 (95)
Non‐White	7 (5)
Ethnicity, *n* (%)	
Non‐Hispanic	121 (92)
Hispanic	10 (8)
Stage at diagnosis, *n* (%)	
I	25 (19)
II	45 (34)
III	62 (47)
Tumor location, *n* (%)	
Colon	85 (64)
Rectum	47 (36)
Neoadjuvant treatment, *n* (%)	
No	101 (77)
Yes	31 (23)
Tumor grade, *n* (%)	
Grade 1: Well differentiated	23 (17)
Grade 2: Moderately differentiated	85 (64)
Grade 3: Poorly differentiated	18 (14)
Tumor budding by area (mm^2^)	
Mean ± SD	4.75 ± 4.21
Median (IQR)	3.0 (2.0–7.0)
Tumor budding clinical categories, *n* (%)	
Low (0–4 buds)	77 (58)
Intermediate (5–9 buds)	37 (28)
High (≥ 10 buds)	18 (14)
Body mass index (kg/m^2^), *n* (%)	
Normal weight (≥ 18.5 and < 25 kg/m^2^)	31 (23)
Overweight (≥ 25 and < 30 kg/m^2^)	48 (36)
Obese (≥ 30 kg/m^2^)	52 (39)
Smoking status, *n* (%)	
Non‐smoker	60 (45)
Former smoker	39 (30)
Current smoker	7 (5)
NSAID‐use at least once/week during the past year, *n* (%)	
No	31 (23)
Yes (Aspirin or aspirin plus non‐aspirin)	23 (17)
Yes (non‐aspirin)	33 (25)
Biomarkers of inflammation/angiogenesis/cell‐to‐cell adhesion	
CRP [mg/L]	
Mean ± SD	13.9 ± 41.0
Median (IQR)	4.0 (1.8–10.9)
SAA [mg/L]	
Mean ± SD	33.1 ± 96.3
Median (IQR)	4.9 (2.6–11.9)
IL‐6 [pg/mL]	
Mean ± SD	2.10 ± 2.69
Median (IQR)	1.36 (1.00–2.01)
IL‐8 [pg/mL]	
Mean ± SD	28.0 ± 20.0
Median (IQR)	22.4 (18.4–29.4)
sICAM‐1 [mg/L]	
Mean ± SD	0.44 ± 0.14
Median (IQR)	0.40 (0.35–0.51)
sVCAM‐1 [mg/L]	
Mean ± SD	0.62 ± 0.18
Median (IQR)	0.60 (0.50–0.71)
VEGF‐A [pg/mL]	
Mean ± SD	727 ± 491
Median (IQR)	640 (365–982)
VEGF‐D [pg/mL]	
Mean ± SD	1120 ± 219
Median (IQR)	1133 (980–1268)
TNF‐α [pg/mL]	
Mean ± SD	3.23 ± 1.16
Median (IQR)	3.00 (2.65–3.58)

*Note:* Missing values across the population: Ethnicity: *n* = 1; BMI: *n* = 1; tumor grade: *n* = 6; smoking status: *n* = 26; NSAID‐use: *n* = 45; CRP: *n* = 1; SAA: *n* = 1; IL‐6: *n* = 46; IL‐8: *n* = 45; sICAM‐1: *n* = 1; sVCAM‐1: *n* = 1; VEGF‐A: *n* = 2; VEGF‐D: *n* = 2; TNF‐α: *n* = 45.

Abbreviations: IQR, interquartile range; SD, standard deviation.

Patients' demographic and clinicopathological characteristics by tertiles of tumor budding are shown in Table S1. The distributions of patients' demographic and clinicopathological characteristics did not differ by tumor budding tertiles. Only VEGF‐A levels differed across tumor budding tertiles.

**FIGURE 2:**
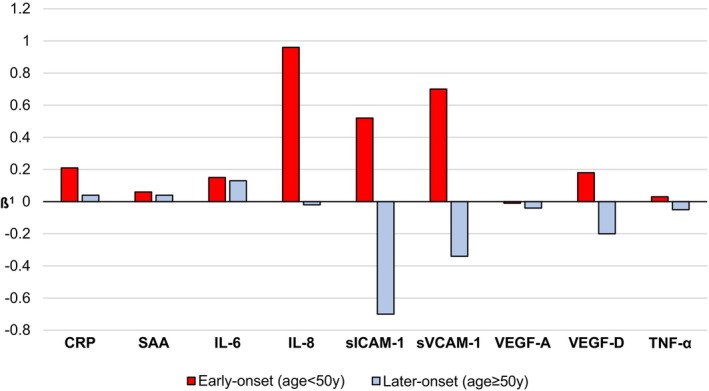
Associations between biomarkers of inflammation/angiogenesis/cell‐to‐cell adhesion and tumor budding in colorectal cancer, stratified by age. (Early‐onset: *N* = 22; Later‐onset: *N* = 110). Due to skewed distributions, biomarker and tumor budding values were log2‐transformed. Missing values across the population: CRP: *N* = 1; SAA: *N* = 1; IL‐6: *N* = 46; IL‐8: *N* = 45; sICAM‐1: *N* = 1; sVCAM‐1: *N* = 1; VEGF‐A: *N* = 2; VEGF‐D: *N* = 2; TNF‐α: *N* = 45; NSAID‐use: *N* = 45. ^1^Standardized regression coefficient *ß* (Model 2: Adjusted for sex, stage, neoadjuvant treatment, NSAIDs).

Table 2 shows the associations between the biomarkers of inflammation, angiogenesis, and cell‐to‐cell adhesion with tumor budding counts. In multivariable models, sICAM‐1 was inversely associated with tumor budding (M1: *β* = −0.57, *p*‐value = 0.03; M2: *β* = −0.59, *p*‐value = 0.03), although the association became non‐statistically significant after correction for multiple testing. In the stratified analyses by age, sICAM‐1 was inversely associated with tumor budding among patients with later‐onset colorectal cancer (Table 3) (M1: *β* = −0.71, *p*‐value = 0.008, *p*‐interaction = 0.046; M2: *β* = −0.70, *p*‐value = 0.01, *p*‐interaction = 0.11), while IL‐8 (M2: *β* = 0.96, *p*‐value = 0.01, *p*‐interaction = 0.34) and sVCAM‐1 (M1: *β* = 1.48, *p*‐value = 0.04, *p*‐interaction = 0.02) showed positive associations with tumor budding count among early‐onset patients (Figure 2). However, these associations became non‐statistically significant after correction for multiple testing. A similar association was found for sICAM‐1 among patients aged ≥ 60 years old (Table S3). In stratified analysis by sex (Table 4), we observed a positive association between CRP and tumor budding count among male patients (M1: *β* = 0.23, *p*‐value = 0.001, *p*‐interaction = 0.002; M2: *β* = 0.22, *p*‐value = 0.004, *p*‐interaction = 0.005), while a negative association was observed for sICAM‐1 among female patients (M1: *β* = −0.81, *p*‐value = 0.03, *p*‐interaction = 0.35). After adjustment for multiple comparisons (Bonferroni adjustment), these associations would not be considered statistically significant. Sensitivity analyses excluding patients who received neoadjuvant treatment did not change study findings (Tables S4–S6).

**TABLE 2:** Multiple linear regression models, testing for associations between biomarkers of inflammation/angiogenesis/cell‐to‐cell adhesion and tumor budding in colorectal cancer (*n* = 132).

	Model 1^a^		Model 2^b^	
	Age‐ and sex‐adjusted		Multivariable adjusted	
	*β*	*p*	*β*	*p*
CRP [mg/L]	0.07	0.15	0.06	0.25
SAA [mg/L]	0.05	0.31	0.04	0.42
IL‐6 [pg/mL]	0.18	0.15	0.10	0.40
IL‐8 [pg/mL]	0.06	0.76	0.06	0.75
sICAM‐1 [mg/L]	−0.57	**0.03**	−0.59	**0.03**
sVCAM‐1 [mg/L]	−0.16	0.55	−0.12	0.65
VEGF‐A [pg/mL]	0.02	0.83	−0.03	0.76
VEGF‐D [pg/mL]	−0.04	0.91	0.02	0.94
TNF‐α [pg/mL]	0.05	0.85	0.04	0.87

*Note:* Due to skewed distributions, biomarker and tumor budding values were log2‐transformed. Missing values across the population: CRP: *n* = 1; SAA: *n* = 1; IL‐6: *n* = 46; IL‐8: *n* = 45; sICAM‐1: *n* = 1; sVCAM‐1: *n* = 1; VEGF‐A: *n* = 2; VEGF‐D: *n* = 2; TNF‐α: *n* = 45; NSAID‐use: *n* = 45. Bolded *p*‐values indicate statistical significance at *p* < 0.05.

^a^ Adjusted for age, sex.

^b^ Adjusted for age, sex, stage, neoadjuvant treatment, NSAIDs.

**TABLE 3:** Multiple linear regression models, testing for associations between biomarkers of inflammation/angiogenesis/cell‐to‐cell adhesion and tumor budding in colorectal cancer, stratified by age (*n* = 132).

Early‐onset (age < 50 y): *n* = 22 (17%) Later‐onset (age ≥ 50 y): *n* = 110 (83%)	Model 1^a^			Model 2^b^		
	Sex‐adjusted			Multivariable adjusted		
	*β*	*p*	*p‐*inter action	*β*	*p*	*p‐*inter action
CRP [mg/L]						
Early‐onset	0.20	0.11	0.24	0.21	0.15	0.27
Later‐onset	0.05	0.38		0.04	0.46	
SAA [mg/L]						
Early‐onset	0.04	0.75	1.00	0.06	0.67	0.80
Later‐onset	0.05	0.35		0.04	0.45	
IL‐6 [pg/mL]						
Early‐onset	0.30	0.23	0.64	0.15	0.59	0.86
Later‐onset	0.15	0.32		0.13	0.40	
IL‐8 [pg/mL]						
Early‐onset	0.67	0.10	0.08	0.96	0.01	0.34
Later‐onset	−0.11	0.59		−0.02	0.94	
sICAM‐1 [mg/L]						
Early‐onset	1.19	0.21	0.046	0.52	0.63	0.11
Later‐onset	−0.71	0.008		−0.70	0.01	
sVCAM‐1 [mg/L]						
Early‐onset	1.48	0.04	0.02	0.70	0.42	0.051
Later‐onset	−0.41	0.15		−0.34	0.25	
VEGF‐A [pg/mL]						
Early‐onset	0.12	0.67	0.68	−0.01	0.99	0.67
Later‐onset	0.00	0.98		−0.04	0.75	
VEGF‐D [pg/mL]						
Early‐onset	1.22	0.21	0.14	0.18	0.89	0.14
Later‐onset	−0.24	0.50		−0.20	0.60	
TNF‐α [pg/mL]						
Early‐onset	0.92	0.12	0.16	0.03	0.96	0.86
Later‐onset	−0.17	0.58		−0.05	0.88	

*Note:* Due to skewed distributions, biomarker and tumor budding values were log2‐transformed. Missing values across the population: CRP: *n* = 1; SAA: *n* = 1; IL‐6: *n* = 46; IL‐8: *n* = 45; sICAM‐1: *n* = 1; sVCAM‐1: *n* = 1; VEGF‐A: *n* = 2; VEGF‐D: *n* = 2; TNF‐α: *n* = 45; NSAID‐use: *n* = 45.

^a^ Adjusted for sex.

^b^ Adjusted for sex, stage, neoadjuvant treatment, NSAIDs.

**TABLE 4:** Multiple linear regression models, testing for associations between biomarkers of inflammation/angiogenesis/cell‐to‐cell adhesion and tumor budding in colorectal cancer, stratified by sex (*n* = 132).

Female: *n* = 58 (44%); Male: *n* = 74 (56%)	Model 1^a^			Model 2^b^		
	Age‐adjusted			Multivariable adjusted		
	*β*	*p*	*p‐*inter action	*β*	*p*	*p‐*inter action
CRP [mg/L]						
Female	−0.06	0.35	0.002	−0.08	0.23	0.005
Male	0.23	0.001		0.22	0.004	
SAA [mg/L]						
Female	−0.03	0.67	0.18	−0.05	0.55	0.23
Male	0.10	0.11		0.08	0.22	
IL‐6 [pg/mL]						
Female	0.08	0.65	0.47	−0.08	0.65	0.30
Male	0.26	0.13		0.23	0.20	
IL‐8 [pg/mL]						
Female	−0.29	0.28	0.12	−0.19	0.42	0.32
Male	0.28	0.26		0.21	0.43	
sICAM‐1 [mg/L]						
Female	−0.81	0.03	0.35	−0.76	0.06	0.56
Male	−0.35	0.34		−0.47	0.21	
sVCAM‐1 [mg/L]						
Female	−0.68	0.08	0.06	−0.55	0.18	0.18
Male	0.29	0.43		0.12	0.75	
VEGF‐A [pg/mL]						
Female	0.04	0.80	0.89	−0.02	0.87	0.83
Male	0.01	0.95		−0.06	0.68	
VEGF‐D [pg/mL]						
Female	−0.10	0.84	0.86	0.04	0.94	0.84
Male	0.02	0.97		0.06	0.90	
TNF‐α [pg/mL]						
Female	−0.58	0.13	0.03	−0.21	0.55	0.17
Male	0.55	0.14		0.38	0.34	

*Note:* Due to skewed distributions, biomarker and tumor budding values were log2‐transformed. Missing values across the population: CRP: *n* = 1; SAA: *n* = 1; IL‐6: *n* = 46; IL‐8: *n* = 45; sICAM‐1: *n* = 1; sVCAM‐1: *n* = 1; VEGF‐A: *n* = 2; VEGF‐D: *n* = 2; TNF‐α: *n* = 45; NSAID‐use: *n* = 45.

^a^ Adjusted for age.

^b^ Adjusted for age, stage, neoadjuvant treatment, NSAIDs.

## 4 Discussion

In this study, we report for the first time on the associations between biomarkers of inflammation, angiogenesis, and cell‐to‐cell adhesion and tumor budding count in colorectal cancer patients. Overall, none of these biomarkers demonstrated statistically significant associations with tumor budding count after correction for multiple testing. In subgroup analyses, we observed patterns where sICAM‐1 was inversely associated with tumor budding among patients with later‐onset colorectal cancer and female patients. CRP was positively associated with tumor budding among male patients, while IL‐8 and sVCAM‐1 were positively associated with tumor budding among patients with early‐onset colorectal cancer. These did not remain statistically significant after correcting for multiple testing.

Elevated tumor budding counts and systemic inflammation are both established adverse prognostic factors in colorectal cancer [4, 5, 6, 7, 12]. The inverse association observed between sICAM‐1 levels and tumor budding count was contrary to our study hypothesis. This unexpected finding may reflect complex tumor–immune interactions, particularly the dynamic role of the inflammatory tumor microenvironment in regulating cell adhesion and tumor progression. However, the association did not retain statistical significance after correction for multiple testing. In preclinical models, epithelial‐to‐mesenchymal transition (EMT), a histopathological feature associated with tumor budding, has been shown to induce sICAM‐1 secretion by cancer cells, promoting angiogenesis, recruiting myeloid cells, and supporting tumor cell proliferation [24]. In clinical studies, a positive correlation between baseline sICAM‐1 concentrations and tumor burden was observed in patients with head and neck squamous cell carcinoma at the start of radio‐chemotherapy [40]. So far, no existing clinical study has examined the role of sICAM‐1 in tumor budding in colorectal cancer patients, and further research is needed to clarify this relationship. Understanding the relationship between systemic inflammation biomarkers and tumor budding may provide important insights for the development of targeted therapies.

In our stratified analyses, evaluating age and sex‐specific differences in the associations between the biomarkers and tumor budding, sICAM‐1 was inversely associated with tumor budding among patients with later‐onset colorectal cancer while IL‐8 and sVCAM‐1 were positively associated with tumor budding among patients with early‐onset colorectal cancer. Additionally, sICAM‐1 was negatively associated with tumor budding among female patients while CRP was positively associated with tumor budding among male patients. However, these associations did not remain statistically significant after correction for multiple testing and, therefore, should be interpreted with caution. C‐reactive protein (CRP) is a well‐established marker of systemic inflammation, widely recognized for its sensitivity in detecting the acute‐phase response [13]. We have previously shown that elevated CRP levels were linked to poorer colorectal cancer prognosis [34]. These patterns may reflect age‐ and sex‐related differences in immune function, tumor biology, or hormonal influences on the inflammatory response. Estrogen has been shown to modulate immune pathways in colorectal cancer, influencing tumor growth, inflammation, and the broader tumor microenvironment. Evidence suggests that estrogen may exert protective effects by promoting a tumor microenvironment that is less conducive to cancer progression, primarily through inhibition of inflammation and reduction of immunosuppressive signaling [41]. This may partly explain the observed inverse relationship between sICAM‐1 and tumor budding among female patients in our study. Conversely, the positive relationship between CRP and tumor budding in male patients is consistent with the known pro‐inflammatory role of CRP. Notably, women tend to have a lower incidence of colorectal cancer than men, supporting the hypothesis that sex hormones, particularly estrogen, may confer protection through modulation of inflammatory and immune responses [42]. Likewise, early‐onset colorectal cancer has been associated with distinct molecular features and heightened immune activation [43]. This study provides the first observation of these patterns and may serve as preliminary data to inform future research examining how inflammatory, angiogenic, and cell‐adhesion biomarkers relate to tumor budding.

Our study has several strengths. To our knowledge, this is the first study to examine associations between a comprehensive panel of biomarkers related to inflammation, angiogenesis, and cell‐to‐cell adhesion and tumor budding count in patients with colorectal cancer, while also exploring potential differences by age and sex. The study employed rigorous and standardized procedures for collecting serum samples, questionnaire data, and clinical information. However, a few limitations should be noted. First, while our study represents the first report of these associations, the observed relationships between biomarkers and tumor budding, including the age‐ and sex‐specific interactions, were not statistically significant after correction for multiple testing. These findings may serve as pilot observations to guide future studies aimed at further investigating these potential relationships and elucidating the underlying biological mechanisms. Second, the generalizability of our findings is limited, as the study population was predominantly White. Future studies with larger and more diverse cohorts are needed to examine these associations across different racial and ethnic groups and to explore potential population‐specific differences. Another limitation of our study is its cross‐sectional design. Since both biomarker levels and tumor budding were measured at baseline, causality cannot be inferred, and a temporal sequence cannot be established. Biomarker levels may have been influenced by the tumor and/or treatment itself, potentially leading to reverse causation. Longitudinal studies are needed to better understand the directionality and underlying mechanisms of these associations. Lastly, tumor budding involves subjective interpretation of complex morphological patterns, which can vary even among experts. Hence, we performed extensive quality control and adjusted for scoring pathologists in our statistical analyses.

## 5 Conclusion

In conclusion, this study did not observe significant associations between biomarkers of inflammation, angiogenesis, and cell‐to‐cell adhesion and tumor budding count in colorectal cancer patients after multiple testing correction. Some of the suggested findings may serve as pilot observations to guide future studies in larger, diverse cohorts investigating the biological mechanisms underlying tumor budding and their potential relevance to colorectal cancer prognosis.

## Author Contributions

**Oda Hausmann:** conceptualization, formal analysis, investigation, methodology, software, visualization, writing – original draft, writing – review and editing. **Pauline P. Schobert:** formal analysis, methodology, investigation, writing – original draft, writing – review and editing. **Jennifer Ose:** funding acquisition, investigation, project administration, supervision, writing – original draft, writing – review and editing. **Caroline Himbert:** data curation, investigation, writing – original draft, writing – review and editing. **Maria Pletneva:** data curation, investigation, writing – review and editing. **Jolanta Jedrzkiewicz:** data curation, investigation, project administration, supervision, writing – review and editing. **Anne Nguyen:** data curation, formal analysis, investigation, writing – review and editing. **Tengda Lin:** data curation, formal analysis, investigation, methodology, software, writing – review and editing. **Christy A. Warby:** data curation, investigation, project administration, supervision, writing – review and editing. **Sheetal Hardikar:** investigation, writing – review and editing. **Anita R. Peoples:** investigation, writing – review and editing. **Ildiko Strehli:** investigation, writing – review and editing. **Lyen C. Huang:** data curation, investigation, writing – review and editing. **Jessica N. Cohan:** data curation, investigation, writing – review and editing. **Bartley Pickron:** data curation, investigation, writing – review and editing. **Courtney Scaife:** data curation, investigation, writing – review and editing. **Christopher I. Li:** funding acquisition, investigation, writing – review and editing. **William M. Grady:** investigation, writing – review and editing. **David Shibata:** funding acquisition, investigation, writing – review and editing. **Adetunji T. Toriola:** funding acquisition, investigation, writing – review and editing. **Martin Schneider:** funding acquisition, investigation, writing – review and editing. **Jane C. Figueiredo:** funding acquisition, investigation, writing – review and editing. **Erin M. Siegel:** funding acquisition, investigation, writing – review and editing. **Biljana Gigic:** funding acquisition, investigation, writing – review and editing. **Stephan Herzig:** investigation, project administration, supervision, writing – review and editing. **Mmadili N. Ilozumba:** conceptualization, formal analysis, investigation, methodology, project administration, software, supervision, writing – original draft, writing – review and editing. **Cornelia M. Ulrich:** conceptualization, funding acquisition, investigation, project administration, resources, supervision, writing – original draft, writing – review and editing.

## Ethics Statement

The study received approval from the Institutional Review Board at the University of Utah under IRB #77147.

## Consent

All participants granted their written informed consent.

## Conflicts of Interest

C.M.U. has as cancer center director oversight over research funded by several pharmaceutical companies but has not received funding directly herself. W. M. Grady is a scientific advisory board member for Freenome, Guardant Health, and SEngine and consultant for DiaCarta, Natera, Guidepoint and GLG. He receives research support from LucidDx.

## Supporting information


**Table S1:** Baseline demographic and clinicopathologic characteristics by tumor budding tertiles of individuals with primary invasive colorectal cancer (*n* = 132).
**Table S2:** Baseline demographic and clinicopathologic characteristics by tumor budding in clinical categories of individuals with primary invasive colorectal cancer (*n* = 132).
**Table S3:** Multiple linear regression models, testing for associations between biomarkers of inflammation/angiogenesis/cell‐to‐cell adhesion and tumor budding in colorectal cancer, stratified by age (*n* = 132).
**Table S4:** Sensitivity analysis—multiple linear regression models, testing for associations between biomarkers of inflammation/angiogenesis/cell‐to‐cell adhesion and tumor budding in colorectal cancer excluding patients with neoadjuvant treatment (*n* = 101).
**Table S5:** Sensitivity analysis—multiple linear regression models, testing for associations between biomarkers of inflammation/angiogenesis/cell‐to‐cell adhesion and tumor budding in colorectal cancer excluding patients with neoadjuvant treatment, stratified by age (*n* = 101).
**Table S6:** Sensitivity analysis—multiple linear regression models, testing for associations between biomarkers of inflammation/angiogenesis/cell‐to‐cell adhesion and tumor budding in colorectal cancer excluding patients with neoadjuvant treatment, stratified by sex (*n* = 101).

## Data Availability

The ColoCare Study data is available from colocarestudy_admin@hci.utah.edu on reasonable request and as described on the ColoCare website (https://uofuhealth.utah.edu/huntsman/labs/colocare‐consortium/). Our data‐sharing procedures are available online (https://uofuhealth.utah.edu/huntsman/labs/colocare‐consortium/data‐sharing/new‐projects.php). For questions, please contact colocarestudy_admin@hci.utah.edu. Further information is available from the corresponding author upon request.
