# Supplementary material for: Associations of Biomarkers of Systemic Inflammation, Angiogenesis, and Cell‐to‐Cell Adhesion With Tumor Budding Among Early‐Onset and Later‐Onset Colorectal Cancer Patients
Source: Cancer Med. 2025 Sep 23;14(18):e71267. doi: 10.1002/cam4.71267 (PMC12455364; doi:10.1002/cam4.71267)
Supplement: Supplementary file 1 — Table S1: Baseline demographic and clinicopathologic characteristics by tumor budding tertiles of individuals with primary invasive colorectal cancer (n = 132). Table S2: Baseline demographic and clinicopathologic characteristics by tumor budding in clinical categories of individuals with primary invasive colorectal cancer (n = 132). Table S3: Multiple linear regression models, testing for associations between biomarkers of inflammation/angiogenesis/cell‐to‐cell adhesion and tumor budding in colorectal cancer, stratified by age (n = 132). Table S4: Sensitivity analysis—multiple linear regression models, testing for associations between biomarkers of inflammation/angiogenesis/cell‐to‐cell adhesion and tumor budding in colorectal cancer excluding patients with neoadjuvant treatment (n = 101). Table S5: Sensitivity analysis—multiple linear regression models, testing for associations between biomarkers of inflammation/angiogenesis/cell‐to‐cell adhesion and tumor budding in colorectal cancer excluding patients with neoadjuvant treatment, stratified by age (n = 101). Table S6: Sensitivity analysis—multiple linear regression models, testing for associations between biomarkers of inflammation/angiogenesis/cell‐to‐cell adhesion and tumor budding in colorectal cancer excluding patients with neoadjuvant treatment, stratified by sex (n = 101). [file CAM4-14-e71267-s001.docx]

**Supplementary Tables 1-6:**

| Supplementary Table 1: Baseline demographic and clinicopathologic characteristics by tumor budding tertiles of individuals with primary invasive colorectal cancer (n=132) | | | | | |
| --- | --- | --- | --- | --- | --- |
|  | | **Tumor budding tertiles** | |  | |
| Characteristics | **Study Population** | **T1 (0-2 buds)** | **T2 (>2–6 buds)** | **T3 (>6 buds)** | ***p-*value**^1^ |
| Total, n (%) | 132 (100) | 47 (36) | 43 (33) | 42 (32) |  |
| Age (years) | | | | | |
| Mean ± SD  Median (IQR) | 61 ± 13  61 (52-71) | 60 ± 14  61 (52-71) | 59 ± 13  61 (51-67) | 62 ± 12  63 (53-72) | 0.62 |
| Age, n (%) | | | | | |
| Early-onset (<50y)  Later-onset ($\boldsymbol{\geq}$50y) | 22 (17)  110 (83) | 9 (19)  38 (81) | 7 (16)  36 (84) | 6 (14)  36 (86) | 0.83 |
| Sex, n (%) | | | | | |
| Female  Male | 58 (44)  74 (56) | 23 (49)  24 (51) | 20 (47)  23 (53) | 15 (36)  27 (64) | 0.42 |
| Race, n (%) | | | | | |
| White  Non-White | 125 (95)  7 (5) | 45 (96)  2 (4) | 41 (95)  2 (5) | 39 (93)  3 (7) | 0.81 |
| Stage at diagnosis, n (%) | | | | | |
| I  II  III | 25 (19)  45 (34)  62 (47) | 13 (28)  18 (38)  16 (34) | 7 (16)  14 (33)  22 (51) | 5 (12)  13 (31)  24 (57) | 0.18 |
| Tumor location, n (%) | | | | | |
| Colon  Rectum | 85 (64)  47 (36) | 28 (60)  19 (40) | 30 (70)  13 (30) | 27 (64)  15 (36) | 0.60 |
| Neoadjuvant treatment, n (%) | | | | | |
| No  Yes | 101 (77)  31 (23) | 33 (70)  14 (30) | 35 (81)  8 (19) | 33 (79)  9 (21) | 0.43 |
| Tumor grade, n (%) | | | | | |
| Grade 1  Grade 2  Grade 3 | 23 (18)  85 (67)  18 (14) | 8 (19)  31 (72)  4 (9) | 7 (17)  25 (61)  9 (22) | 8 (19)  29 (69)  5 (12) | 0.55 |
| Body mass index ^2^ (kg/m^2^), n (%) | | | | | |
| Normal weight  Overweight  Obese | 31 (24)  48 (37)  52 (40) | 13 (28)  16 (34)  18 (38) | 10 (23)  13 (30)  20 (47) | 8 (20)  19 (46)  14 (34) | 0.54 |
| Smoking Status, n (%) | | | | | |
| Non-smoker  Former smoker  Current smoker | 60 (57)  39 (37)  7 (7) | 22 (54)  17 (41)  2 (5) | 20 (63)  9 (28)  3 (9) | 18 (55)  13 (39)  2 (6) | 0.77 |
| NSAID-use at least once/week in the past year ^3^, n (%) | | | | | |
| No  Yes (Aspirin)  Yes (non-aspirin) | 31 (36)  23 (26)  33 (38) | 16 (46)  11 (31)  8 (23) | 7 (27)  5 (19)  14 (54) | 8 (31)  7 (27)  11 (42) | 0.16 |
|  |  |  |  |  |  |
| Biomarker | **Study Population** | **T1** | **T2** | **T3** | ***p-*value**^1^ |
|  | | | | | ***p-*trend** |
| CRP [mg/L] | | | | | |
| Mean ± SD  Median (IQR) | 2.06 ± 2.09  2.00 (0.88-3.45) | 1.59 ± 1.81  1.77 (0.14-2.75) | 2.50 ± 2.22  2.35 (1.20-3.65) | 2.11 ± 2.19  1.99 (0.48-4.01) | 0.12 |
|  |  |  |  |  | 0.35 |
| SAA [mg/L] | | | | | |
| Mean ± SD  Median (IQR) | 2.81 ± 2.14  2.29 (1.38-3.57) | 2.38 ± 1.80  1.96 (1.33-3.25) | 3.07 ± 2.41  2.34 (1.64-3.73) | 3.01 ± 2.18  2.43 (1.38-4.33) | 0.24 |
|  |  |  |  |  | 0.22 |
| IL-6 [pg/mL] | | | | | |
| Mean ± SD  Median (IQR) | 0.63 ± 0.96  0.45 (0.00-1.01) | 0.32 ± 0.73  0.35 (-0.25-0.61) | 0.82 ± 0.89  0.67 (0.09-1.39) | 0.65 ± 1.13  0.43 (-0.09-0.99) | 0.17 |
|  |  |  |  |  | 0.41 |
| IL-8 [pg/mL] | | | | | |
| Mean ± SD  Median (IQR) | 4.62 ± 0.66  4.48 (4.20-4.88) | 4.56 ± 0.63  4.43 (4.21-4.85) | 4.64 ± 0.78  4.48 (4.07-4.91) | 4.64 ± 0.55  4.60 (4.27-4.86) | 0.88 |
|  |  |  |  |  | 0.73 |
| sICAM-1 [mg/L] | | | | | |
| Mean ± SD  Median (IQR) | -1.25 ± 0.39  -1.33 (-1.50-[-0.98]) | -1.15 ± 0.40  -1.18 (-1.40-[-0.92]) | -1.28 ± 0.40  -1.40 (-1.55-[-1.01]) | -1.32 ± 0.36  -1.34 (-1.53-[-1.13]) | 0.10 |
|  |  |  |  |  | 0.053 |
| sVCAM-1 [mg/L] | | | | | |
| Mean ± SD  Median (IQR) | -0.73 ± 0.38  -0.74 (-1.00-[-0.50]) | -0.72 ± 0.43  -0.69 (-1.03-[-0.50]) | -0.72 ± 0.41  -0.81 (-1.00-[-0.39]) | -0.75 ± 0.30  -0.72 (-0.99-[-0.55]) | 0.94 |
|  |  |  |  |  | 0.74 |
| VEGF-A [pg/mL] | | | | | |
| Mean ± SD  Median (IQR) | 9.18 ± 1.01  9.32 (8.51-9.94) | 9.10 ± 0.97  9.07 (8.42-9.94) | 9.50 ± 0.92  9.65 (8.78-10.11) | 8.95 ± 1.08  9.16 (8.38-9.62) | 0.04 |
|  |  |  |  |  | 0.36 |
| VEGF-D [pg/mL] | | | | | |
| Mean ± SD  Median (IQR) | 10.1 ± 0.3  10.1 (9.9-10.3) | 10.1 ± 0.3  10.1 (9.9-10.4) | 10.1 ± 0.2  10.1 (9.9-10.3) | 10.1 ± 0.3  10.2 (10.0-10.3) | 0.79 |
|  |  |  |  |  | 0.64 |
| TNF-α [pg/mL] | | | | | |
| Mean ± SD  Median (IQR) | 1.62 ± 0.45  1.58 (1.40-1.84) | 1.59 ± 0.43  1.57 (1.29-1.75) | 1.66 ± 0.48  1.59 (1.43-1.84) | 1.59 ± 0.45  1.58 (1.38-1.84) | 0.80 |
|  |  |  |  |  | 0.88 |
| Due to skewed distributions, biomarker values were log2-transformed. | | | | | |
| Missing values across the population: BMI: n=1; tumor grade: n=6; smoking status: n=26; NSAID-use: n=45; CRP: n=1; SAA: n=1; IL-6: n=46; IL-8: n=45; sICAM-1: n=1; sVCAM-1: n=1; VEGF-A: n=2; VEGF-D: n=2; TNF-α: n=45 | | | | | |
| Abbreviations: IQR= interquartile range; SD= standard deviation | | | | | |
| ^1^ ANOVA (continuous outcomes) or chi-square test of independence (categorical outcomes) *p*-values testing differences between tumor budding tertiles  ^2^ Normal weight (≥18.5 and <25 kg/m^2^); Overweight (≥25 and <30 kg/m^2^); Obese (≥30 kg/m^2^)  ^3^ No; Yes (Aspirin or aspirin plus other NSAIDs than aspirin); Yes (other NSAIDs than aspirin) | | | | | |

| Supplementary Table 2: Baseline demographic and clinicopathologic characteristics by tumor budding in clinical categories of individuals with primary invasive colorectal cancer (n=132) | | | | | |
| --- | --- | --- | --- | --- | --- |
|  | | **Tumor budding clinical categories^1^** | | |  |
| Characteristics | **Study Population** | **Low** | **Intermediate** | **High** | ***p-*value**^2^ |
| Total, n (%) | 132 (100) | 77 (58) | 37 (28) | 18 (14) |  |
| Age (years) | | | | |  |
| Mean ± SD  Median (IQR) | 61 ± 13  61 (52-71) | 60 ± 14  61 (52-70) | 60 ± 11  59 (52-69) | 64 ± 12  65 (56-73) | 0.53 |
| Age, n (%) | | | | |  |
| Early-onset (<50y)  Later-onset ($\boldsymbol{\geq}$50y) | 22 (17)  110 (83) | 13 (17)  61 (83) | 6 (16)  31 (84) | 3 (17)  15 (83) | 1.00 |
| Sex, n (%) | | | | |  |
| Female  Male | 58 (44)  74 (56) | 37 (48)  40 (52) | 14 (38)  23 (62) | 7 (39)  11 (61) | 0.53 |
| Race, n (%) | | | | |  |
| White  Non-White | 125 (95)  7 (5) | 74 (96)  3 (4) | 34 (92)  3 (8) | 17 (94)  1 (6) | 0.64 |
| Stage at diagnosis, n (%) | | | | |  |
| I  II  III | 25 (19)  45 (34)  62 (47) | 19 (25)  28 (36)  30 (39) | 3 (8)  11 (30)  23 (62) | 3 (17)  6 (33)  9 (50) | 0.14 |
| Tumor location, n (%) | | | | |  |
| Colon  Rectum | 85 (64)  47 (36) | 49 (64)  28 (36) | 24 (65)  13 (35) | 12 (67)  6 (33) | 0.97 |
| Neoadjuvant treatment, n (%) | | | | |  |
| No  Yes | 101 (77)  31 (23) | 58 (75)  19 (25) | 29 (78)  8 (22) | 14 (78)  4 (22) | 0.93 |
| Tumor grade, n (%) | | | | |  |
| Grade 1  Grade 2  Grade 3 | 23 (18)  85 (67)  18 (14) | 14 (19)  47 (65)  11 (15) | 7 (19)  25 (69)  4 (11) | 2 (11)  13 (72)  3 (17) | 0.90 |
| Body mass index ^3^ (kg/$\mathbf{m}^{\mathbf{2}}$), n (%) | | | | |  |
| Normal weight  Overweight  Obese | 31 (24)  48 (37)  52 (40) | 20 (26)  27 (35)  30 (39) | 9 (24)  11 (30)  17 (46) | 2 (12)  10 (59)  5 (29) | 0.30 |
| Smoking Status, n (%) | | | | |  |
| Non-smoker  Former smoker  Current smoker | 60 (57)  39 (37)  7 (7) | 39 (62)  21 (33)  3 (5) | 12 (44)  11 (41)  4 (15) | 9 (56)  7 (44)  0 (0) | 0.22 |
| NSAID-use at least once/week in the past year ^4^, n (%) | | | | |  |
| No  Yes (Aspirin)  Yes (non-aspirin) | 31 (36)  23 (26)  33 (38) | 21 (39)  15 (28)  18 (33) | 5 (24)  6 (29)  10 (48) | 5 (42)  2 (17)  5 (42) | 0.65 |
|  | | | | | |
| Biomarker | **Study Population** | **Low** | **Intermediate** | **High** | ***p*-value^2^** |
|  | | | | | ***p*-trend** |
| CRP [mg/L] | | | | | |
| Mean ± SD  Median (IQR) | 2.06 ± 2.09  2.00 (0.88-3.45) | 1.86 ± 2.00  1.93 (0.79-2.86) | 2.09 ± 2.22  2.00 (0.63-3.70) | 2.82 ± 2.16  2.30 (1.20-4.70) | 0.22 |
|  |  |  |  |  | 0.10 |
| SAA [mg/L] | | | | | |
| Mean ± SD  Median (IQR) | 2.81 ± 2.14  2.29 (1.38-3.57) | 2.71 ± 2.04  2.30 (1.47-3.37) | 2.61 ± 2.21  2.08 (1.16-2.93) | 3.65 ± 2.31  3.02 (1.69-5.82) | 0.20 |
|  |  |  |  |  | 0.21 |
| IL-6 [pg/mL] | | | | | |
| Mean ± SD  Median (IQR) | 0.63 ± 0.96  0.45 (0.00-1.01) | 0.59 ± 0.88  0.45 (0.03-0.94) | 0.43 ± 0.74  0.36 (-0.16-0.90) | 1.18 ± 1.43  0.65 (0.28-1.19) | 0.21 |
|  |  |  |  |  | 0.19 |
| IL-8 [pg/mL] | | | | | |
| Mean ± SD  Median (IQR) | 4.62 ± 0.66  4.48 (4.20-4.88) | 4.64 ± 0.75  4.46 (4.19-4.88) | 4.64 ± 0.63  4.57 (4.18-5.06) | 4.49 ± 0.31  4.51 (4.23-4.74) | 0.74 |
|  |  |  |  |  | 0.55 |
| sICAM-1 [mg/L] | | | | | |
| Mean ± SD  Median (IQR) | -1.25 ± 0.39  -1.33 (-1.50-[-0.98]) | -1.22 ± 0.40  -1.30 (-1.49-[-0.95]) | -1.27 ± 0.38  -1.33 (-1.49-[-1.01]) | -1.31 ± 0.39  -1.37 (-1.56-[-1.13]) | 0.59 |
|  |  |  |  |  | 0.30 |
| sVCAM-1 [mg/L] | | | | | |
| Mean ± SD  Median (IQR) | -0.73 ± 0.38  -0.74 (-1.00-[-0.50]) | -0.74 ± 0.41  -0.73 (-1.05-[-0.49]) | -0.69 ± 0.32  -0.74 (-0.93[-0.54]) | -0.78 ± 0.37  -0.81 (-1.01-[-0.53]) | 0.74 |
|  |  |  |  |  | 0.94 |
| VEGF-A [pg/mL] | | | | | |
| Mean ± SD  Median (IQR) | 9.18 ± 1.01  9.32 (8.51-9.94) | 9.17 ± 0.97  9.22 (8.42-9.94) | 9.24 ± 1.09  9.39 (8.66-9.99) | 9.10 ± 1.06  9.32 (8.35-9.68) | 0.88 |
|  |  |  |  |  | 0.97 |
| VEGF-D [pg/mL] | | | | | |
| Mean ± SD  Median (IQR) | 10.1 ± 0.3  10.1 (9.9-10.3) | 10.1 ± 0.3  10.1 (9.9-10.3) | 10.1 ± 0.3  10.2 (10.0-10.3) | 10.0 ± 0.4  10.1 (9.9-10.3) | 0.60 |
|  |  |  |  |  | 0.62 |
| TNF-α [pg/mL] | | | | | |
| Mean ± SD  Median (IQR) | 1.62 ± 0.45  1.58 (1.40-1.84) | 1.61 ± 0.43  1.56 (1.36-1.79) | 1.60 ± 0.52  1.58 (1.43-1.82) | 1.69 ± 0.38  1.63 (1.47-1.84) | 0.81 |
|  |  |  |  |  | 0.65 |
| Due to skewed distributions, biomarker values were log2-transformed. | | | | | |
| Missing values across the population*:* BMI: n=1; tumor grade: n=6; smoking status: n=26; NSAID-use: n=45; CRP: n=1; SAA: n=1; IL-6: n=46; IL-8: n=45; sICAM-1: n=1; sVCAM-1: n=1; VEGF-A: n=2; VEGF-D: n=2; TNF-α: n=45 | | | | | |
| Abbreviations: IQR= interquartile range; SD= standard deviation | | | | | |
| ^1^ Low (0-4 tumor buds); Intermediate (5-9 tumor buds); High (≥10 buds)  ^2^ ANOVA (continuous outcomes) or chi-square test of independence (categorical outcomes) *p*-values testing differences between tumor budding clinical categories  ^3^ Normal weight (≥18.5 and <25 kg/m^2^); Overweight (≥25 and <30 kg/m^2^); Obese (≥30 kg/m^2^)  ^4^ No; Yes (Aspirin or aspirin plus other NSAIDs than aspirin); Yes (other NSAIDs than aspirin) | | | | | |

| **Supplementary Table 3: Multiple linear regression models, testing for associations between biomarkers of inflammation/angiogenesis/cell-to-cell adhesion and tumor budding in colorectal cancer, stratified by age. (n=132)** | | | | | | |
| --- | --- | --- | --- | --- | --- | --- |
|  | ***Model 1 ^1^***  ***Sex-adjusted*** | | | ***Model 2 ^2^***  ***Multivariable adjusted*** | | |
| Age<60years: n=61 (46)  Age≥60years: n=71 (54) | **β** | ***p-value*** | ***p-*inter**  **action** | **β** | ***p-value*** | ***p-*inter**  **action** |
| **CRP [mg/L]** | | | | | | |
| Age<60years  Age≥60years | 0.10  0.03 | 0.09  0.71 | 0.44 | 0.09  0.01 | 0.12  0.89 | 0.46 |
| **SAA [mg/L]** | | | | | | |
| Age<60years  Age≥60years | 0.04  0.06 | 0.54  0.39 | 0.84 | 0.02  0.06 | 0.73  0.41 | 0.71 |
| **IL-6 [pg/mL]** | | | | | | |
| Age<60years  Age≥60years | 0.24  0.09 | 0.12  0.70 | 0.59 | 0.13  0.09 | 0.45  0.69 | 0.79 |
| **IL-8 [pg/mL]** | | | | | | |
| Age<60years  Age≥60years | 0.21  -0.13 | 0.37  0.65 | 0.35 | 0.19  0.06 | 0.44  0.86 | 0.86 |
| **sICAM-1 [mg/L]** | | | | | | |
| Age<60years  Age≥60years | -0.22  -0.80 | 0.59  0.02 | 0.25 | -0.26  -0.94 | 0.51  0.01 | 0.45 |
| **sVCAM-1 [mg/L]** | | | | | | |
| Age<60years  Age≥60years | -0.20  -0.07 | 0.56  0.87 | 0.81 | -0.06  0.00 | 0.85  1.00 | 0.65 |
| **VEGF-A [pg/mL]** | | | | | | |
| Age<60years  Age≥60years | 0.02  0.03 | 0.91  0.80 | 0.90 | -0.13  0.02 | 0.43  0.89 | 0.77 |
| **VEGF-D [pg/mL]** | | | | | | |
| Age<60years  Age≥60years | -0.04  -0.01 | 0.94  0.98 | 0.98 | -0.20  0.01 | 0.73  0.99 | 0.88 |
| **TNF-α [pg/mL]** | | | | | | |
| Age<60years  Age≥60years | 0.61  -0.32 | 0.13  0.40 | 0.13 | 0.39  0.16 | 0.36  0.70 | 0.77 |
| Due to skewed distributions, biomarker and tumor budding values were log2-transformed. | | | | | | |
| **Missing values across the population:** CRP: n=1; SAA: n=1; IL-6: n=46; IL-8: n=45; sICAM-1: n=1; sVCAM-1: n=1; VEGF-A: n=2; VEGF-D: n=2; TNF-α: n=45; NSAID-use: n=45 | | | | | | |
| **^1^** adjusted for sex  **^2^** adjusted for sex, stage, neoadjuvant treatment, NSAIDs | | | | | | |

| **Supplementary Table 4:** **Sensitivity Analysis** – **Multiple linear regression models, testing for associations between biomarkers of inflammation/angiogenesis/cell-to-cell adhesion and tumor budding in colorectal cancer excluding patients with neoadjuvant treatment. (n=101)** | | | | |
| --- | --- | --- | --- | --- |
|  | ***Model 1^1^***  ***Age- and sex-adjusted*** | | ***Model 2^2^***  ***Multivariable adjusted*** | |
|  | **β** | ***p-value*** | **β** | ***p-value*** |
| **CRP [mg/L]** | 0.04 | 0.39 | 0.03 | 0.61 |
| **SAA [mg/L]** | 0.06 | 0.24 | 0.05 | 0.31 |
| **IL-6 [pg/mL]** | 0.17 | 0.17 | 0.14 | 0.29 |
| **IL-8 [pg/mL]** | -0.13 | 0.50 | -0.13 | 0.53 |
| **sICAM-1 [mg/L]** | -0.47 | 0.07 | -0.43 | 0.11 |
| **sVCAM-1 [mg/L]** | -0.25 | 0.35 | -0.18 | 0.50 |
| **VEGF-A [pg/mL]** | 0.06 | 0.57 | -0.01 | 0.95 |
| **VEGF-D [pg/mL]** | -0.04 | 0.92 | -0.09 | 0.80 |
| **TNF-α [pg/mL]** | -0.37 | 0.19 | -0.32 | 0.28 |
| Due to skewed distributions, biomarker and tumor budding values were log2-transformed. | | | | |
| **Missing values across the population:** CRP: n=1; SAA: n=1; IL-6: n=37; IL-8: n=37; sICAM-1: n=1; sVCAM-1: n=1; VEGF-A: n=1; VEGF-D: n=1; TNF-α: n=37; NSAID-use: n=31 | | | | |
| **^1^** adjusted for age, sex  **^2^** adjusted for age, sex, stage, NSAIDs | | | | |

| **Supplementary Table 5: Sensitivity Analysis – Multiple linear regression models, testing for associations between biomarkers of inflammation/angiogenesis/cell-to-cell adhesion and tumor budding in colorectal cancer excluding patients with neoadjuvant treatment, stratified by age. (n=101)** | | | | | | |
| --- | --- | --- | --- | --- | --- | --- |
|  | ***Model 1^1^***  ***Sex-adjusted*** | | | ***Model 2^2^***  ***Multivariable adjusted*** | | |
| Early-onset (age<50y): n=10 (10%)  Later-onset (age≥50y): n=91 (90%) | **β** | ***p-*value** | ***p-*inter action** | **β** | ***p-*value** | ***p-*inter action** |
| **CRP [mg/L]** | | | | | | |
| Early-onset  Later-onset | 0.17  0.03 | 0.34  0.54 | 0.47 | 0.18  0.02 | 0.36  0.73 | 0.55 |
| **SAA [mg/L]** | | | | | | |
| Early-onset  Later-onset | 0.09  0.05 | 0.57  0.31 | 0.82 | 0.24  0.04 | 0.07  0.43 | 0.62 |
| **IL-6 [pg/mL]** | | | | | | |
| Early-onset  Later-onset | 0.15  0.20 | 0.44  0.17 | 0.65 | 0.30  0.15 | -  0.32 | 0.73 |
| **IL-8 [pg/mL]** | | | | | | |
| Early-onset  Later-onset | 0.17  -0.13 | 0.81  0.53 | 0.92 | 0.55  -0.10 | -  0.66 | 0.78 |
| **sICAM-1 [mg/L]** | | | | | | |
| Early-onset  Later-onset | 0.53  -0.51 | 0.74  0.054 | 0.46 | -0.27  -0.49 | 0.94  0.08 | 0.37 |
| **sVCAM-1 [mg/L]** | | | | | | |
| Early-onset  Later-onset | 1.52  -0.36 | 0.16  0.20 | 0.11 | -1.14  -0.28 | 0.13  0.32 | 0.30 |
| **VEGF-A [pg/mL]** | | | | | | |
| Early-onset  Later-onset | 0.04  0.06 | 0.91  0.57 | 0.97 | 0.38  0.01 | 0.07  0.95 | 0.82 |
| **VEGF-D [pg/mL]** | | | | | | |
| Early-onset  Later-onset | 2.09  -0.29 | 0.08  0.42 | 0.03 | 0.02  -0.29 | 0.98  0.43 | 0.12 |
| **TNF-α [pg/mL]** | | | | | | |
| Early-onset  Later-onset | -0.14  -0.40 | 0.79  0.22 | 0.93 | 0.39  -0.28 | -  0.41 | 0.80 |
| Due to skewed distributions, biomarker and tumor budding values were log2-transformed. | | | | | | |
| **Missing values across the population:** CRP: n=1; SAA: n=1; IL-6: n=37; IL-8: n=37; sICAM-1: n=1; sVCAM-1: n=1; VEGF-A: n=1; VEGF-D: n=1; TNF-α: n=37; NSAID-use: n=31 | | | | | | |
| **^1^** adjusted for sex  **^2^** adjusted for sex, stage, NSAIDs | | | | | | |
| -, sample size was too small, hence no *p*-value was calculated | | | | | | |

| **Supplementary Table 6: Sensitivity Analysis - Multiple linear regression models, testing for associations between biomarkers of inflammation/angiogenesis/cell-to-cell adhesion and tumor budding in colorectal cancer excluding patients with neoadjuvant treatment, stratified by sex. (n=101)** | | | | | | |
| --- | --- | --- | --- | --- | --- | --- |
|  | ***Model 1^1^***  ***Age-adjusted*** | | | ***Model 2^2^***  ***Multivariable adjusted*** | | |
| Female: n=48 (48%)  Male: n=53 (52%) | **β** | ***p*-value** | ***p-*inter action** | **β** | ***p*-value** | ***p-*inter action** |
| **CRP [mg/L]** | | | | | | |
| Female  Male | -0.05  0.18 | 0.44  0.01 | 0.02 | -0.06  0.19 | 0.36  0.01 | 0.02 |
| **SAA [mg/L]** | | | | | | |
| Female  Male | -0.05  0.14 | 0.51  0.02 | 0.053 | -0.07  0.14 | 0.34  0.02 | 0.04 |
| **IL-6 [pg/mL]** | | | | | | |
| Female  Male | 0.07  0.29 | 0.74  0.09 | 0.34 | -0.12  0.31 | 0.53  0.08 | 0.18 |
| **IL-8 [pg/mL]** | | | | | | |
| Female  Male | -0.48  0.08 | 0.14  0.73 | 0.13 | -0.08  -0.04 | 0.80  0.89 | 0.22 |
| **sICAM-1 [mg/L]** | | | | | | |
| Female  Male | -0.75  -0.21 | 0.051  0.57 | 0.31 | -0.53  -0.15 | 0.21  0.70 | 0.41 |
| **sVCAM-1 [mg/L]** | | | | | | |
| Female  Male | -0.53  0.06 | 0.18  0.88 | 0.28 | -0.30  0.11 | 0.46  0.77 | 0.42 |
| **VEGF-A [pg/mL]** | | | | | | |
| Female  Male | -0.05  0.14 | 0.79  0.28 | 0.43 | -0.12  0.13 | 0.46  0.36 | 0.32 |
| **VEGF-D [pg/mL]** | | | | | | |
| Female  Male | 0.16  -0.26 | 0.77  0.56 | 0.44 | 0.04  -0.06 | 0.93  0.90 | 0.47 |
| **TNF-α [pg/mL]** | | | | | | |
| Female  Male | -0.71  0.03 | 0.07  0.94 | 0.20 | -0.21  0.09 | 0.59  0.84 | 0.37 |
| Due to skewed distributions, biomarker and tumor budding values were log2-transformed. | | | | | | |
| **Missing values across the population:** CRP: n=1; SAA: n=1; IL-6: n=37; IL-8: n=37; sICAM-1: n=1; sVCAM-1: n=1; VEGF-A: n=1; VEGF-D: n=1; TNF-α: n=37; NSAID-use: n=31 | | | | | | |
| **^1^** adjusted for age  **^2^** adjusted for age, stage, NSAIDs | | | | | | |
